# Time to and Predictors of CD4+ T-Lymphocytes Recovery in HIV-Infected Children Initiating Highly Active Antiretroviral Therapy in Ghana

**DOI:** 10.1155/2011/896040

**Published:** 2011-05-11

**Authors:** Lorna Renner, Meghan Prin, Fang-Yong Li, Bamenla Goka, Veronika Northrup, Elijah Paintsil

**Affiliations:** ^1^Department of Child Health, University of Ghana Medical School, Accra, Ghana; ^2^University of Medicine and Dentistry of New Jersey, Piscataway, NJ 08854-8021, USA; ^3^Yale Center for Analytical Sciences, Yale University School of Medicine, New Haven, CT 06510-3206, USA; ^4^Departments of Pediatrics and Pharmacology, Yale University School of Medicine, 333 Cedar Street, New Haven, CT 06510-3206, USA

## Abstract

*Background*. CD4+ T-lymphocyte monitoring is not routinely available in most resource-limited settings. We investigated predictors of time to CD4+ T-lymphocyte recovery in HIV-infected children on highly active antiretroviral (HAART) at Korle-Bu Teaching Hospital, Ghana. *Methods*. Time to CD4+ T-lymphocyte recovery was defined as achieving percent CD4+ T-lymphocytes of 25%. We used Cox proportional hazard models for identifying significant predictor variables. *Results*. Of the 233 children with complete CD4+ T-lymphocyte data, the mean age at HAART initiation was 5.5 (SD = 3.1) years. The median recovery time was 60 weeks (95% CL: 55–65). Evidence at baseline of severe suppression in CD4+ T-lymphocyte count adjusted for age, age at HAART initiation, gender, and having parents alive were statistically significant in predicting time to CD4+ T-lymphocyte recovery. *Conclusions*. A targeted approach based on predictors of CD4+ T-lymphocyte recovery can be a viable and cost-effective way of monitoring HAART in HIV-infected children in resource-limited settings.

## 1. Introduction

HIV primarily targets CD4+ T-lymphocytes, and recent studies have shown that during primary HIV infection, HIV viral replication in CD4+ T-lymphocytes in gut-associated lymphoid tissue (GALT) results in significant CD4+ T-lymphocyte depletion [1, 2]. Chronic HIV infection affects both quantitative and qualitative function of CD4+ T-lymphocytes, and disease progression results from continuous depletion of these cells with a concomitant increase in risk for opportunistic infections, acquired immune deficiency syndrome (AIDS), and death [3–5]. Given the central role of CD4+ T-lymphocytes in HIV pathogenesis, CD4+ T-lymphocyte determination during the course of HIV disease is one of the most reliable predictors of prognosis [6]. 

The therapeutic goal of highly active antiretroviral therapy (HAART) is to suppress HIV viral replication and restore immune function (i.e., CD4+ T-lymphocyte recovery). The standard of care for monitoring treatment in HIV-infected children is the routine laboratory monitoring of CD4+ T-lymphocyte percentage or count and HIV viral load at least every 3-4 months [6]. However, CD4+ T-lymphocyte and viral load testing, for monitoring the efficacy of HAART, are not routinely available in most resource-limited settings [7]. Though several low-cost and technically less complex devices have been developed for CD4+ T-lymphocyte testing, there are still quite a number of centers in resource-limited countries with no access to reliable CD4+ T-lymphocyte testing [8, 9]. Furthermore, the cost of commercially available viral load testing is prohibitive (between $50 and $100 per test), making it unaffordable and inaccessible to many HIV treatment centers in resource-limited countries [10].

Several studies have demonstrated that the efficacy of HAART in HIV-infected children in resource-limited countries is comparable to that of children in resource-rich countries [11–19]. Given the empirical obstacles of using the standard of care for monitoring HAART therapy in resource-limited countries and the need to develop clinical practices that would reduce the overall cost of patient care, we investigated factors affecting treatment response among HIV-positive children on HAART in a resource-limited country. In particular, we examined the predictors of the time to CD4+ T-lymphocyte recovery using retrospective longitudinal data.

## 2. Methods

### 2.1. Study Cohort

This was a single center retrospective study at the Pediatric HIV/AIDS Care program at Korle-Bu Teaching Hospital in Accra, Ghana. The study population consisted of all HIV-infected children on HAART, since pediatric antiretroviral therapy became available in 2004. All the patients included in the analysis were on their first-line regimen of nonnucleoside analog-based HAART consisting of zidovudine (AZT) or stavudine (d4T) plus lamivudine (3TC), plus either nevirapine (NVP) or efavirenz (EFV). We analyzed longitudinal data extracted from medical records of children on treatment with at least two CD4+ T-lymphocyte determinations between June 2004 and December 2009. The study was reviewed and approved by the Ethics and Protocol Review Committees of University of Ghana Medical School and Yale School of Medicine.

The main study outcome was time to CD4+ T-lymphocyte recovery, defined as achieving and maintaining a target CD4 percentage of 25% after initiation of HAART. Because of well-known large natural decline and variation in absolute CD4+ T-lymphocyte numbers in early childhood, the percentage of CD4+ T-lymphocytes is preferentially used in the management of pediatric HIV infection; the target is to achieve at least 25% of CD4+ T-lymphocytes according to age on treatment [20, 21].

The predictor variables included gender, age at start of therapy, WHO clinical staging and WHO immune classification at baseline, mode of transmission, HIV and living status of parents, TB diagnosis and treatment, being a graduate of prevention of mother-to-child transmission (PMTCT) program, and adherence to treatment regimen. Since 2004, CD4+ T-lymphocyte counts and percentages were done at least every 6 months. CD4+ T-lymphocyte count and percentage were quantified by a dual-platform flow cytometry technology using an FACSCount system (Becton-Dickinson, Franklin Lakes, NJ) at the clinical laboratory at Korle-Bu Teaching Hospital according to manufacturer's instructions. Samples were processed within four hours. The laboratory is certified by the South African Public Health Reference Laboratory and they participate in an external quality assurance testing program by the South African Public Health Reference Laboratory. Viral load testing is not available routinely. 

### 2.2. Statistical Analysis

Patient characteristics were summarized with *N* and percentage. Kaplan-Meier approach was used to describe the cumulative proportion of CD4+ T-lymphocyte recovery as of particular time in the followup. We used (1–Survival) to plot the trajectories of recovery function over time. The median recovery time across the categories of specific patient characteristics was evaluated with the log-rank test. We used unadjusted Cox proportional hazards (CPHs) models to evaluate independent associations between each of the patient characteristics and the rate of CD4+ T-lymphocyte recovery. The magnitudes of the associations were summarized with hazard ratios (HRs) and 95% confidence intervals (95% CI). Adjusted associations were obtained from multivariate CPH. Significance was established with alpha of 0.05. All analyses were performed with SAS 9.2 (Cary, NC).

## 3. Results

### 3.1. Characteristics of Study Population

Three hundred and fifty-one HIV-infected children were started on HAART between 2004 and 2009 at the Pediatric HIV clinic at Korle-Bu Teaching Hospital. During the study period, 233 of the children on HAART had at least two CD4+ T-lymphocyte counts and were included in our analysis. The followup time ranged from 11 to 349 weeks, with the median followup of 127 weeks. There were two deaths; one died after meeting the study's primary outcome (i.e., CD4+ T-lymphocyte recovery), and the other died after 54 weeks without recovery. Both patients were included in the analysis.  Table 1 illustrates the clinical and demographic characteristics of study participants. About 96% of the children on HAART were a year of age or older, with an almost equal male-to-female ratio. The mean age at initiation of HAART was 5.5 (SD = 3.1) years, and the majority were started on HAART at an advanced stage of their disease, corroborated by their WHO clinical staging and immune classification. About half of the cohort had been diagnosed and treated for tuberculosis. Of note is the small percentage (1.3%) of PMTCT graduates who became infected with HIV and went on to HAART treatment.

The baseline CD4+ T-lymphocyte count of children between one and five years old was significantly higher than those older than six years (*P* < .0001). The median baseline CD4+ T-lymphocyte count was 423 (IQR: 272–742), 531 (IQR: 305–774), and 174 cells/mm^3^ (IQR: 35–371) for children less than one year old, between one and five, and six and older, respectively.

### 3.2. Time to CD4+ T-Lymphocyte Recovery

One hundred and seventy-two (73.8%) of study subjects achieved immunological recovery, that is, achieved a target CD4+ T-lymphocyte percentage of 25%, during the study period. In the unadjusted analyses, the median recovery time for all ages was 60 weeks (95% CL: 55–65). Children between ages one and five years recovered faster, with median recovery time of 52 weeks (95% CL: 38–60 weeks), followed by children less than one year old (median: 66 weeks; 95% CL: 19–210 weeks), and then children six years or older (median: 69 weeks; 95% CL: 57–84 weeks). The median recovery time was shorter for children with no or moderate evidence of immune suppression at the start of HAART therapy and at least one parent alive (Table 2). We also observed trends for the following predictors of faster CD4+ T-lymphocyte recovery: female gender (*P* = .06) and both parents with HIV positive status (.10). 

**Figure 1:**
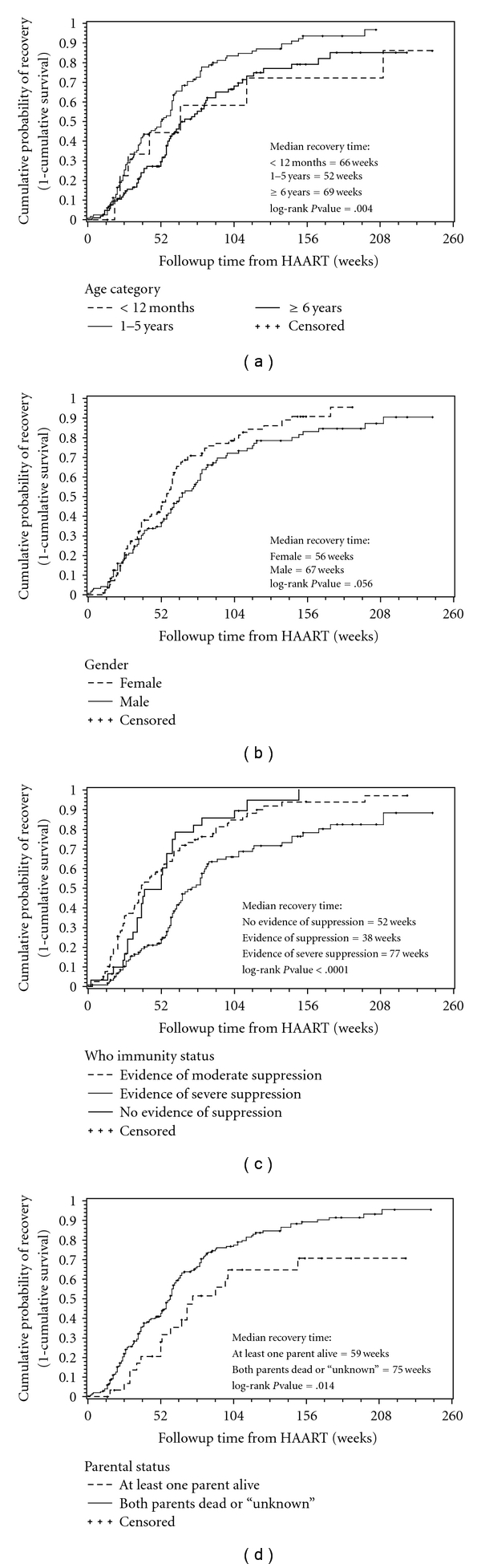
Kaplan-Meier survival curves of CD4+ T-lymphocyte recovery versus significant predictor variables of recovery for the 233 HIV-infected children on highly active antiretroviral therapy (HAART) from 2004 to 2009. (a) Age category: the median recovery time for ages <12 months, 1 to 6 years, and ≥6 years was 66, 52, and 69 weeks, respectively (logrank,  *P* = .004). (b) Gender: the median recovery time for female and male was 58 and 67 weeks, respectively (logrank, *P* = .056). (c) WHO immune classification: the median recovery time for no evidence of suppression, evidence of moderate suppression, and evidence of severe suppression was 52, 38, and 77 weeks, respectively (logrank, *P* = .004). (d) Parental living status: the median recovery time for at least one parent alive and both parents dead or “unknown” was 59 and 75 weeks, respectively (logrank, *P* = .014).

Kaplan-Meier curves of the cumulative probability over time of CD4+ T-lymphocyte recovery stratified by age category at start of HAART, gender, WHO immune classification, and parental living status are illustrated in Figure 1.

### 3.3. Predictors of Time to Recovery of CD4+ T-Lymphocyte

In unadjusted analyses, starting HAART between 1–5 years of age (HR = 1.64, *P* = .002), higher baseline CD4+ T-lymphocyte count (HR = 1.05 per 100-cell increase,  *P* = .001), and having at least one parent alive (HR = 1.82, *P* = .016) were significantly associated with faster CD4+ T-lymphocyte recovery. We found a border line association between female gender and time to recovery (HR = 1.34, *P* = .059). There was no statistically significant association between mode of HIV transmission, parental HIV status, WHO clinical staging, treatment adherence self-report, previous TB diagnosis and treatment, and CD4+ T-lymphocyte recovery (Table 3). 

Because baseline CD4+ T-lymphocyte was significantly associated with age at the start of HAART therapy and since WHO immune classification at the start of HAART incorporates the effect of child's age, we retained WHO immune classification in the multivariate model instead of baseline CD4+ T-lymphocytes. In a multivariate model (Table 4), statistically significant predictor variables of time to recovery were parental living status (*P* = .025), gender (*P* = .051), age at start of HAART (*P* = .014), and WHO immune classification at start of HAART (*P* < .0001). The probability of recovering faster was two times greater in children with at least one parent alive than those with both parents deceased (HR: 2.00, 95% CI: 1.18, 3.38). Females were 1.37 times more likely to achieve faster recovery than males (HR: 1.37, 95% CI: 1.00, 1.87). Children who were started on HAART between one and 5 years of age had 1.59 times greater rate of recovery than children 6 years of age or older.

## 4. Discussion

Our findings corroborate with earlier, though few and far in between, reports on the efficacy of NNRTI-based HAART regimens in HIV-infected children in resource-limited countries. Moreover, we identified predictor variables that could be useful in developing a targeted approach to CD4+ testing and cost-effective monitoring of HAART with CD4+ T-lymphocyte counts where resources are limited. Furthermore, children with correlated variables predictive of slow CD4+ T-lymphocyte recovery could be identified and followed more frequently and carefully.

 Seventy-four percent of study participants achieved immunological recovery during the study followup period of mean duration of 110 (SD = 67.7)  weeks. The magnitude of CD4+ T-lymphocyte recovery is consistent with other reports on pediatric antiretroviral therapy in resource-limited countries [11, 12, 16, 22, 23]. In a study from Thailand with a median followup of 168 weeks, the probability of achieving CD4% of greater or equal to 25% ranged from 65% to 98% in children with baseline CD4% ranging from ≤5% to 24% [14]. In a report on efficacy of HAART in 14 resource-limited countries (including 10 from sub-Saharan Africa), 62% of children attained CD4 of ≥25% after one year of receiving antiretroviral therapy [23]. In a cohort study from Cambodia, median CD4% rose from 6% at baseline to 25% at 12 months [16]. In contrast, the PATCG -219 study reported that only 26% to 49% of children on HAART with baseline CD4% ranging from <5 to 24% achieved CD4%  >2 after three years on therapy [24, 25]. Moreover, the time to CD4+ T-lymphocyte recovery in our cohort (the median recovery was 60 weeks) was shorter than most of the reports from resource-rich countries but compared favorably with that of studies from other resource-limited countries. 

Although studies from sub-Saharan Africa have demonstrated the efficacy of HAART in HIV-infected children, the predictors of immunological response have not been investigated systematically [26]. In most centers, CD4+ T-lymphocyte monitoring is either unavailable or done infrequently due to resource constraints. We found it expedient for the same reasons to look at patient characteristics that could predict immunological success in a resource-limited setting. Statistically significant predictor variables of CD4+ T-lymphocyte recovery in our study were age at starting HAART, gender, WHO immune classification, and parental living status. WHO clinical staging, mode of transmission, HIV status of parents, TB diagnosis and treatment, and adherence to treatment regimen were not statistically significant predictors. Being a graduate of the Korle-Bu Teaching Hospital PMTCT program was a significant predictor of time to recovery; however, this finding was based on a very small sample (*N* = 3). It is worth mentioning that the very few numbers of HIV-infected children from the program attest to the merit of the PMTCT programs. One can speculate that the faster recovery rate of graduates of PMTCT program who go on HAART may be due to good followup and strong patient compliance. 

Our finding that children aged 1–5 years at the start of HAART achieve CD4+ T-lymphocyte recovery significantly faster than older children confirms the results from previous pediatric studies in both resource-limited and resource-rich countries [14, 24, 27]. We also found that gender is an indicator of faster CD4+ T-lymphocyte recovery, which is consistent with a recent Thai study that reported female children having a better immunologic and virologic response than males [14]. There are no reports from sub-Saharan Africa on association between immune recovery and gender in pediatrics, with the exception of one study that found male gender to be associated with virologic failure [28]. The reasons for this gender effect are not well understood. Interestingly, in HIV-infected adults, gender differences in treatment outcome (i.e., immunologic and virologic) have received mixed reviews [29, 30]. 

Consistent with previous reports from resource-rich settings [19, 31–33] and resource-limited countries [14, 28], baseline absolute CD4+ T-lymphocyte count correlated positively with the rate of immune reconstitution. HIV-infected children in the United States who began antiretroviral therapy when severely immunosuppressed (CD4+ T-lymphocyte <15%) had a significantly shorter time to first-line regimen switch than children who were immunocompetent or moderately immunosuppressed at initiation [34]. Combining the effect of age and baseline CD4+ T-lymphocyte on recovery raises the importance of early diagnosis and treatment to achieve immune reconstitution and preservation of first-line regimens. This is particularly pertinent to resource-limited countries where second-line regimes are limited and expensive. 

Having at least one parent alive was found to predict rate of immune recovery. This relationship may be rather complex and there may be other contributing factors, considering the extended family structure and support in Africa. Interestingly, a study from Cambodia found that children who were orphaned were more likely to fail therapy, and the authors attributed this to poor adherence to treatment [16]. Among children with complete information on parental HIV status (67%), having both HIV-positive parents contributed to faster recovery than having one parent with HIV, albeit statistically not significant (*P* = .13). We are inclined to infer that this is really a surrogate for having one versus both parents alive, hence the stronger observed association of parental living status with the outcome in our study. 

CD4+ T-lymphocyte count is a significant parameter in monitoring ART in HIV-infected individuals [6, 35–37]. In most resource-limited countries, treatment failure is defined by immunologic criteria due to the lack of routine viral load monitoring. Unfortunately, not all HIV centers have access to CD4+ T-lymphocyte monitoring due to operational constraints. Despite that, recent studies in children from resource-limited countries have demonstrated that immunologic and virologic outcomes of antiretroviral therapy is comparable to that of children in resource-rich countries [11–18]. In these studies, on average the baseline CD4+ T-lymphocyte count doubled after six months on therapy. The rate of increase remained the same over the second 6 months and slowed thereafter. In one study, there was no significant increase in CD4+ T-lymphocyte count at 12 and 24 months [18]. This is consistent with studies in HIV-infected adults where CD4+ T-lymphocyte recovery plateaued after 4 to 5 years of HAART despite complete viral suppression [25, 33, 38]. From these studies, one can surmise that frequent (e.g., every three to four months) determination of CD4+ T-lymphocyte count may not be necessary. Our findings of predictors of CD4+ T-lymphocyte recovery further argue for the adoption of a more targeted approach or “personalized” CD4+ T-lymphocyte testing, particularly in resource-limited countries, rather than one based on a general time interval. The testing interval could be based on an individual's pre-HAART risk of having a poor immune recovery rate. 

Our study, like other retrospective studies, has inherent limitations and also additional limitations due to operational constraints of resource-limited settings. It is a single center study, and, therefore, one has to be cautious in generalizing our findings. Moreover, data on viral loads were not available, which precluded us from examining the effect of HAART on viral load and the correlation between viral load and CD4+ T-lymphocytes. Our findings have to be validated with effect of HAART on viral load decline, since it is not uncommon to have discordance in the gain of CD4+ T-lymphocytes and decline in viral load. Furthermore, the data on adherence to therapy was by self-report and not validated, so we did not use it in the final model. However, with these caveats, our findings are consistent with previous studies, while our proposal of targeted approach to CD4+ T-lymphocyte testing is worth further investigation and validation.

## 5. Conclusion

Our study demonstrates that a clinically meaningful and robust immune recovery in HIV-infected children on HAART is attainable in resource-limited countries and that there are patient characteristics that can predict time to immune recovery. Based on these predictors, a followup analysis can examine how well we can predict a time to CD4+ T-lymphocyte recovery for an individual child so that a targeted approach to CD4+ T-lymphocyte testing could be adopted as a viable and potentially cost-effective approach in monitoring HAART in HIV-infected children in resource-limited countries.

## Figures and Tables

**Table 1:** Characteristics of 233 HIV-infected children on highly active antiretroviral therapy (HAART) from 2004–2009, Accra, Ghana.

Characteristics	*N* (%)
Age category	
<1 year	10 (4.3)
1–5 years	118 (51.0)
>6 years	105 (45.1)
Gender	
Female	112 (48.1)
Male	121 (51.9)
Immune recovery	
No	61 (26.2)
Yes	172 (73.8)
WHO immune classification	
No evidence of suppression	31 (13.3)
Evidence of moderate suppression	80 (34.3)
Sever suppression	122 (52.4)
WHO clinical staging	
I	33 (14.1)
II	53 (22.7)
III	106 (45.5)
IV	41 (17.6)
Previous TB diagnosis and treatment	
Yes	119 (51.1)
No	114 (48.9)
Graduate of PMTCT program	
Yes	3 (1.3)
No	203 (87.1)
Do not know	27 (11.6)
Mode of transmission	
Vertical	199 (85.4)
Indeterminate	11 (4.7)
Do not know	23 (9.9)
Self report of adherence	
Poor	13 (5.6)
Good	46 (19.8)
Excellent	144 (61.8)
Unknown	30 (12.9)
Parental HIV status	
Both parents HIV-positive	28 (12.0)
Only one parent HIV-positive	128 (54.9)
Both parents unknown status	75 (32.2)
Mother unknown and father HIV-negative	2 (0.9)
Parental living status	
Both parents alive	109 (46.8)
One parent known alive	79 (33.9)
Both parents dead	23 (9.9)
Both parents unknown	8 (3.4)
One parent alive and other unknown	14 (6.0)

**Table 2:** Median CD4+ T-lymphocyte recovery time among 233 HIV-infected children on highly active antiretroviral therapy (HAART) from 2004–2009, Accra, Ghana.

	Median recovery time, weeks (95% CL)	*P* value of Log rank
Age category		.004
<1 year	66 (19, 210)	
1–5 years	52 (38, 60)	
>6 years	69 (57, 84)	
WHO immune classification		<.0001
No evidence of suppression	52 (35, 56)	
Evidence of moderate suppression	38 (32, 53)	
Severe suppression	74 (63, 84)	
Gender		.056
Female	56 (44, 60)	
Male	67 (56, 80)	
Graduate of PMTCT		<.0001
Yes	16 (2, 35)	
No	60 (56, 66)	
Parental living status		.014
At least one parent alive*	59 (52, 62)	
Both parents died or Do not know	75 (53, 150)	
Parental HIV status^¥^		.099
Both parents with HIV	39 (26, 60)	
One parent with HIV	62 (57, 71)	

*Include those with “both parents alive”, “status known one alive”, and “status known one alive and another status unknown”.

^¥^ *N* = 156. Exclude “both parents with unknown HIV status” (*n* = 75) and “mother unknown HIV status and father without HIV” (*n* = 2).

**Table 3:** Unadjusted associations of patient characteristics and time to CD4+ T-lymphocyte recovery among 233 HIV-infected children on highly active antiretroviral therapy (HAART) from 2004–2009, Accra, Ghana.

	*N* (%)^§^	Hazard Ratio	*P*-value
Age category at ARV start			.005
<1 year	10 (4.3)	0.93	.864
1–5 years	118 (51.0)	1.64	.002
>6 years	105 (45.1)	1.00	
preHAART CD4+ counts (per 10^2^)	228 (97.9)	1.05	.001
WHO immune classification at ARV start			<.0001
No evidence of suppression	31 (13.3)	2.22	.0004
Evidence of moderate suppression	80 (34.3)	2.08	<.0001
Severe suppression	122 (52.4)	1.00	
Gender			.059
Female	112 (48.1)	1.34	
Male	121 (51.9)	1.00	
Mode of transmission			.928
Vertical transmission	199 (85.4)	0.88	.715
Unknown	23 (9.9)	0.91	.842
Indeterminate^¶^	11 (4.7)	1.00	
Graduate of PMTCT program			<.0001
Yes	3 (2.2)	4.77^‡^	
No	201 (86.3)	1.00	
Parental living status			.054
Known both alive	109 (46.8)	1.80	.024
Known one alive	79 (33.9)	1.86	.020
Both died or unknown	31 (13.3)	1.00	—
Parental HIV status			.241
Both parents with HIV	28 (12.0)	1.46	.131
One parents known with HIV	128 (54.9)	1.00	.999
Both parents unknown	75 (32.2)	1.00	—
WHO clinical staging at ARV start			.197
I	32 (13.7)	0.98	.95
II	53 (22.7)	0.70	.98
III	106 (45.5)	1.09	.71
IV	41 (17.6)	1.00	
Adherence self report			.078
Poor	13 (5.6)	1.80	.131
Good	46 (19.8)	1.24	.607
Excellent	144 (61.8)	1.00	
Unknown	30 (12.9)		
Previous Tb diagnosis and treatment			.803
Yes	119 (51.1)	0.15	
No	114 (48.9)	1.00	

**^§^**% corresponds to proportion of the 233 subjects. The sum of  % and *N* may not be up to 100% and 233, respectively, due to subjects with “unknown” responses.

^¶^Subjects with history of possible maternal transmission, blood transfusion, or sexual abuse.

^‡^(95% CL: 2.90, 18.36).

**Table 4:** Adjusted associations of patient characteristics and time to CD4+ T-lymphocyte recovery in 233 HIV-infected children on highly active antiretroviral therapy (HAART) from 2004–2009, Accra, Ghana.

Predictors	Hazard ratio (95% CL)	*P*-value
Parental living status		.025
Both parents alive	1.98 (1.18, 3.35)	
One parent known to be alive	2.00 (1.18, 3.38)	
Both parents dead or unknown	1.00	
Gender		.051
Female	1.37 (1.00, 1.87)	
Male	1.00	
Age category		.014
<12 months	0.98 (0.44, 2.20)	
1–5 years	1.59 (1.15, 2.19)	
>6 years	1.00	
<12 months*	0.62 (0.28, 1.37)	
1–5 years	1.00	
WHO immune classification		<.0001
No evidence of suppression	2.31 (1.48, 3.63)	
Evidence of moderate suppression	2.04 (1.46, 2.86)	
Severe suppression	1.00	

*Small sample size in children <12 months.
